# Potential anticancer activities of *Rhus coriaria* (sumac) extract against human cancer cell lines

**DOI:** 10.1042/BSR20204384

**Published:** 2021-05-06

**Authors:** Sami A. Gabr, Ahmad H. Alghadir

**Affiliations:** 1 Rehabilitation Research Chair, College of Applied Medical Sciences, King Saud University, Riyadh, KSA; 2 Department of Anatomy, Faculty of Medicine, Mansoura University, Egypt

**Keywords:** Cancer cell lines, Carbonic anhydrase inhibitors, cytotoxic activity, Medicinal plant, Rhus coriaria L

## Abstract

Therapeutic strategies of plant origin are a better choice as both dietary plant products or its isolated active constituents against the development and progression of cancer. The present study aims to evaluate the anticancer activity of sumac (*Rhus coriaria*) against different human cancer MCF-7, PC-3, and SKOV3 cell lines. In addition, the study tries to explore a prospective mechanism of action, assessment of *in vitro* enzyme-inhibitory capacity of sumac extract against hCA I, II, IX, and XII. In the present study, the potential antitumor effects of sumac (*Rhus coriaria)* were explored in the human cancer cell lines; MCF-7, PC-3, and SKOV3 using *in vitro* assays. Apoptotic, cell survival, ELISA immunoassays were also conducted to reveal the inhibitory effects of sumac extract against hCA I, II, IX, and XII. In addition, both Clioquinol and Acetazolamide (AZM) were used as standards to explore the *in vitro* enzyme-inhibitory capacity of sumac extract against hCA I, II, IX, and XII. The hydro-alcoholic extract of *R. coriaria* (Sumac) was subjected to phytochemical analysis using GC/MS assays. Sumac at non-cytotoxic doses of 50 and 100 µM significantly modulates the growth of the MCF-7, PC-3, and SKOV3 cancer cells with a higher inhibitory effect and selectivity to carbonic anhydrase (CA) isoforms; hCA I, II, hCA IX, and XII. The data showed that sumac at doses of 50 and 100 µM significantly inhibited the growth, proliferation, and viability of cancer cells by activating the apoptotic process via caspase-3 overexpression and the regulation of Bcl-2 anti-apoptotic protein.

## Introduction

Cancer is the most common disease that is causing a growing health problem globally. It greatly affects millions of people and significantly continues to increase rapidly for the following years [1].

A steady decline in overall deaths was reported by approx. 1.5% per year among cancer patients following early diagnosis and treatment interventions [2]. Although current therapeutic strategies are efficient, a transient durability effect was reported during the recurrence of cancer. Thus, growing interest was focused on herbal remedies and nutrition as alternative approaches in treating cancer, particularly to breast cancer (BC) patients [3]. Dietary plant products either whole products or isolated active constituents play a potential protective role against the development and progression of cancer disease, including BC [4–8]. In most studies, phytochemicals showed to reduce the growth and progression of cancer via anti-inflammatory, immunomodulatory, and antioxidant activity as well as a modulation of several cellular processes, particularly the proliferation, apoptosis, and angiogenesis of cancer cells [9,10]. Thus, new therapeutic advances that use plant-derived phytochemicals as a source of clinically active anti-cancer agents are highly appreciated [11].

Sumac (*Rhus coriaria L., Anacardiaceae)* used as an alternative medicine for several different diseases [12]. In Middle Eastern cuisine, sumac is commonly used as a sour spice [13,14]. Sumac extracts have used in the treatment of several human diseases [15–18], particularly in cancer [19]. Active compounds present in sumac such as flavonoids, tannins and xanthons [20,21], reveal its potency as antiviral, antimicrobial, anticancer, antioxidant and radical scavenging activities [15–19].

Human carbonic anhydrase (CA) isoforms showed to be highly expressed in many types of tumors. Approx. 15 isoforms of CA were expressed during the progression and growth of the tumors, the most important CA isoforms were the transmembrane tumor-associated isoforms (hCA IX and hCA XII) that significantly overexpressed during proliferation of cancer cells [22,23]. Many chemotherapeutic agents targeted hCA IX and hCA XII isoforms for the treatment of cancer [22–24].

Acetazolamide (AZM), one of the sulfonamide compounds reported as CA inhibitors (CAIs), has a high affinity to bind with human CA isoforms [25]. Recently, CAIs of the sulfonamide origin have profound antitumor effects which significantly proceeds via inhibition of hypoxia-inducible isoforms CA IX and XII, overexpressed in many hypoxic tumors [26]. In addition, Clioquinol (5-chloro-7-iodo-8-hydroxyquinoline; CQ) has been recognized as a novel anticancer drug that is able to disrupt proteasome activity [27–30]. The cytotoxicity of Clioquinol was revealed in several cancer models including leukemia, multiple myeloma, and cancer of prostate, bladder, and breast [28–30]. Clioquinol has been demonstrated to induce cancer cell death via several mechanisms including inhibition of lysosome, NF-κB, histone deacetylases, and mTOR signaling pathway [31–35]. Investigation of CQ has also been extended to study its efficacy as effective of phenolic compound used as CAI. Clioquinol showed to be the best phenol inhibitor against all CA isozymes, with inhibition constants in the range of 3.3–16.0 lM [36]. However, little or no data are known about using herbal based trials particularly sumac or its related phytoconstituents as selective hCA IX and hCA XII inhibitors.

Thus, in the current study, the potential antitumor effects of sumac (*Rhus coriaria)* were explored in the human cancer cell lines using *in vitro* assays. Apoptotic and cell survival assays were also conducted to reveal the inhibitory effects of sumac extract against hCA I, II, IX, and XII. In addition, both Clioquinol and Acetazolamide were used as standards to explore the *in vitro* enzyme-inhibitory capacity of sumac extract against hCA I, II, IX, and XII.

## Materials and methods

The proposal of the current study was approved by the Ethics Committee of the Experimental Animal Care Society, Rehabilitation Research Chair (RRC), College of Applied Medical Sciences, King Saud University, Riyadh, Saudi Arabia, under file number ID: RRC-2019-085.

### Chemicals

All chemicals used were of analytical reagent grade. Acetonitrile and methanol used for HPLC analysis were of Sigma grade (Dublin, Ireland). All chemicals used in the present study are of analytical grade.

### *R. coriaria* plant extraction

Sumac samples were purchased from local practitioner market in Riyadh city, KSA and identified by the Pharmacognosy Laboratory, Pharmacy College, King Saud University. The samples of air-dried sumac fruit (50 g) were treated with 16 ml of the sonicated hydro-alcoholic buffer (EtOH/H_2_O; 70:20) as previously reported in the literature [21]. The extract was subjected to several centrifugation processes, the supernatant collected, and the solvent evaporated under vacuum to produce pure extract deposits. These deposits were re-dissolved with 0.5 ml of EtOH/H_2_O (70:30, v/v) and filtered within a 0.22-µm syringe filter. The final extract residues stored at −20°C until reuse [21].

### GC/MS analysis of sumac phenolic compounds

In this experiment, active constituents were separated and quantified in the concentrated organic buffers by using UltraFast TRACE GC (Thermo Scientific, Co.) [37,38]. In addition, a triple quadrupole mass spectrometer (Waltham, MA, U.S.A.), equipped with a Phenomenex Zebron ZB-5MS (5 m × 0.25 mm i.d. × 0.25 µm film thickness or equivalent) column (411 Madrid Avenue, Torrance, CA, U.S.A.) was used in this protocol to separate and quantify sumac phenolic compounds [37,38].

###  CA inhibition assay

In this experiment, the inhibition of CA isoforms was estimated by using an Applied Photophysics stopped flow instrument as previously reported [24]. In this method, the CA catalyzed CO_2_ hydration activity and Phenol Red (at a concentration of 0.2 mM) has been used as an indicator with 20 mM Hepes (pH 7.5) as buffer, and 20 mM Na_2_SO_4_ (for maintaining constant ionic strength). Then, CA enzyme isoforms were incubated with different concentrations of the sumac extracts (10, 25, 50, 100 μg/ml) for 15 min at room temperature or 4ºC. In addition, both Clioquinol and Acetazolamide were applied as standard inhibitors of CA enzyme isoforms. Finally, the inhibition rates of sumac extracts to CA isoforms were measured at the absorbance maximum of 557 nm and compared with applied standard inhibitors [39,40]. The inhibition constants of each sumac concentration were estimated by using anon-linear least square methods with a PRISM program as previously reported [39,40].

### Cell cultures

Human tumor breast cell lines *(MCF-7*), prostate adenocarcinoma (PC-3), and ovary adenocarcinoma (SKOV3) human cell lines were purchased from the American Type Culture Collection (ATCC, U.S.A.). The cells were cultured in Dulbecco’s modified Eagle’s medium (DMEM, Thermo Fisher Scientific). Then, the cells were incubated at 37°C in a humidified CO_2_ (5%) incubator in flasks supplied with fetal bovine serum (FBS; 10% v/v), 1% antibiotics (100 U/ml penicillin and 100 μg/ml streptomycin) [41–46]. The cells at 80% confluence were subcultured into 96-well plates, 6-well plates, and 25 cm^2^ flasks and were performed in triplicates according to designed experiments previously mentioned [41–46].

### Anti-cancer activity

#### MTT cytotoxicity assay

In the present study, the cytotoxic activity of *R. coriaria* (sumac) extract was measured *in vitro* by using different human cancer cell lines; MCF-7, PC-3, and SKOV3 as previously reported [24,45,46]. The reduction of 3-(4,5-Dimethylthiazol-2-yl)-2,5-diphenyltetrazolium bromide (MTT) into formazan crystal in MCF-7, PC-3, and SKOV3 cells was determined to find out the potential cytotoxicity of sumac and related Clioquinol and Acetazolamide as standard inhibitors of CA enzyme isoforms in MCF-7, PC-3, and SKOV3 cells. Human cancer cells; MCF-7, PC-3, and SKOV3 (1 × 10^4^ per well) were cultured in 96-culture plate and exposed to different concentrations of sumac and related standard inhibitors (0, 10, 25, 50, and 100 μg/ml) for 24 h. After 24 h, culture media with tested sumac and standard were discarded from 96-well plates and new culture media containing MTT powder (5 mg/ml) were filled (100 μl/well) and incubated for 4 h at 37°C. The produced formazan crystal was dissolved in dimethyl sulfoxide (DMSO) and optical density (OD) was determined at 570 nm using a microplate reader (Synergy-H1; BioTek, Winooski, VT, U.S.A.).

#### Lactate dehydrogenase enzyme assay

The activity of sumac extract was performed by using lactate dehydrogenase enzyme (LDH) cytotoxicity ELISA (601170 Cayman Chemical Kit, 1180 E. Ellsworth Rd, Ann Arbor, MI, U.S.A.). In this experiment, human target cancer cells are cultured with different concentrations of sumac extract (0, 10, 25, 50, and 100 μg/ml) to induce cell death and subsequent release of LDH to the culture medium. In addition, 10% of Triton X-100 solution provided in the test as negative control. The supernatant containing LDH was transferred to a new wells of ELISA plate and mixed with the LDH reaction solution, and incubated for 30 min in room temperature. For each sumac concentration, the corresponding absorbance was estimated by using ELISA plate reader at 490 nm (A490). Finally, the cytotoxic activity of sumac was calculated as a percentage of the total amount of LDH contained with the target human cancer cells as described by manufacturer.

#### Apoptotic assay

##### Evaluation of Caspase-3

Caspase-3 enzymes as significant biological apoptotic parameters were estimated in sumac-treated and non-treated human cancer cells as previously reported [47]. In this experiment, Bio-Vision colorimetric assay kits were used to estimate the expression of Caspase-3 enzymes in both sumac treated and non-treated human cancer cells (MCF-7, PC-3, and SKOV3). The cells exposed to different concentrations of sumac extract, Clioquinol and Acetazolamide inhibitors of CA enzyme isoforms. In each sample, a spectrophotometer with a 100-µl micro quartz cuvette (Sigma) was used to estimate the concentration of Caspase-3 enzymes at 400 or 405 nm.

##### Evaluation of Bcl-2

Immunoassay techniques were performed to estimate Bcl-2 concentrations using commercially available ELISA kits (Cat# QIA23, Oncogene Research Products, Germany) [48]. In this experiment, Bcl-2 concentrations were estimated in sumac treated and non-treated human cancer cells (MCF-7, PC-3, and SKOV3) and compared with the results of both Clioquinol and Acetazolamide inhibitors of CA enzyme isoforms.

### Statistical analysis

All the assays were conducted in triplicate, and three different microplate wells were used for each concentration. A linear regression analysis was performed to calculate IC_50_ values. Data from the experiments were statistically analyzed by one-way analysis of variance (ANOVA) followed by a post hoc Dunnett’s test using SPSS statistics version 17.0 for Windows (SPSS Inc. 233, Chicago, IL 60606–6412, U.S.A.). *P*-value <0.05 was considered statistically very significant.

## Results

### GC/MS analysis of sumac phytochemical compounds

**Figure 1:**
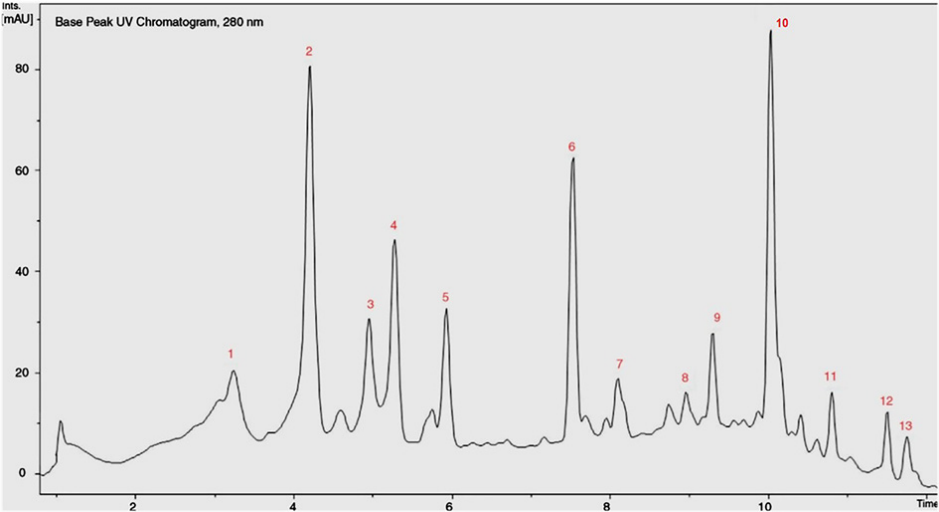
GC/MS analysis of the major identified phenolic compounds in *R. coriaria* extract The identified compounds ordered according to their elution time: (1) Gallic acid, (2) Quercitrin, (3) Isohyperoside, (4) Myricetin glucuronide, (5) Myricetin rutinoside, (6) Dihydroxy-methyl xanthone, (7) β-Sitosterol-hexoside, (8) α-Tocopherol, (9) Linoleic acid, (10) Gallicin, (11) Glucogallic acid, (12) Tri-galloyl-hexoside, and (13) Penta-galloyl-hexoside.

**Figure 2:**
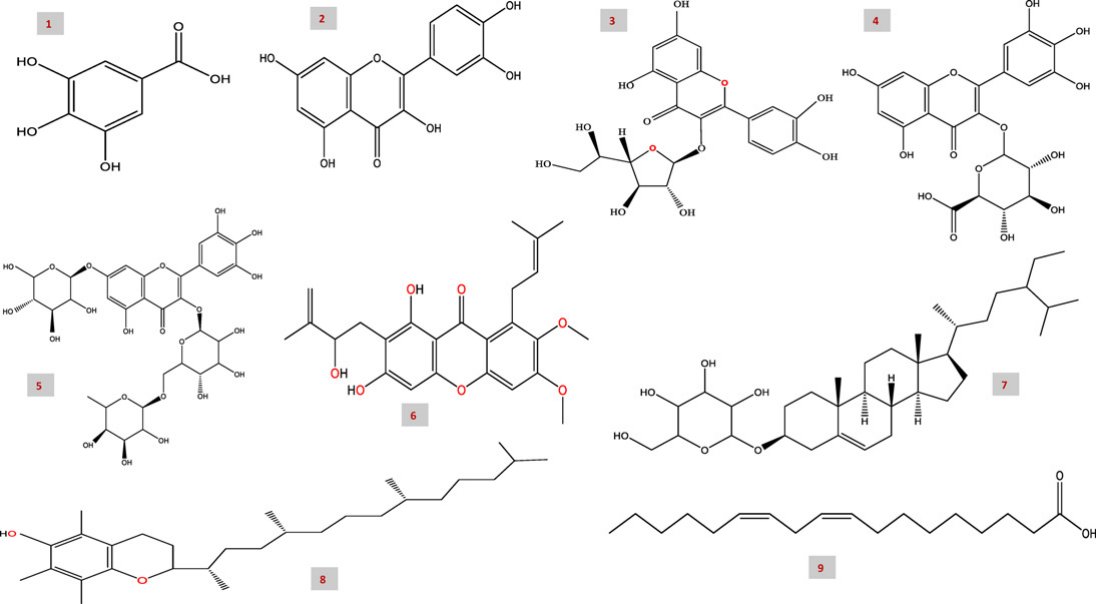
Chemical structures of the identified phenolic compounds in *R. coriaria* extract (1) Gallic acid, (2) Quercitrin, (3) Isohyperoside, (4) Myricetin glucuronide, (5) Myricetin rutinoside, (6) Dihydroxy-methyl xanthone, (7) β-Sitosterol-hexoside, (8) α-Tocopherol, and (9) Linoleic acid.

A potential nine bioactive compounds were estimated in the hydro-alcoholic extract of sumac (Table 1, Figures 1 and 2). In this experiment, gallic acid, isohyperoside, dihydroxy methyl xanthone, β-Sitosterol-hexoside, α-Tocopherol, and linoleic acid were identified at their respective retention times (RTs) in the sumac fruit extract as shown in (Figure 1 and Table 1). In addition, quercitrin (RT; 5.39), myricetin glucuronide (RT; 7.73), and myricetin rutinoside (RT: 9.35) were identified as flavonoid derivatives in the sumac fruit extract as in (Table 1 and Figure 1). There was significant variability in the chemical formula of the identified as shown in (Figure 2). These compounds proposed to potentially involved in the sumac effect on cancer cells and inhibition activity to CA isoforms of the selected human cancer cell lines MCF-7, PC-3, and SKOV3.

**Table 1:** List of identified phenolic compounds in *R. coriaria* extract (RCE) by using GC/MS analysis

Peak	RT (min)	UV max	m/z [M-H] ^(1)^	MS^2^ fragments	Chemical formula
1	4.20	220–295	169.0	125.0	Gallic acid [C_6_H_2_(OH)_3_COOH]
2	5.39	260–357	447.1	301.0	Quercitrin [C_15_H_10_O_7_]
3	5.91	257–358	463.2	301.0	Isohyperoside [C_21_H_2_OO_12_]
4	7.73	261–353	493.1	317.0	Myricetin glucuronide [C_21_H_18_O_14_]
5	9.35	365	625.1	317.0	Myricetin rutinoside [C_33_H_40_O_22_]
6	10.2	220	241.1	195.2	Dihydroxy methyl xanthone [C_25_H_28_O_7_]
7	10.85	204–231	575.1	413.7	β-Sitosterol-hexoside [C_29_H_50_O]
8	11.52	295	429.6	280	α-Tocopherol [C_29_H_50_O_2_]
9	11.86	215.0	279.1	280	Linoleic acid [C_18_H_32_O_2_]

1 In the negative ion detection mode. Abbreviation: RT, retention time.

### *R. coriaria* inhibition activity to CA isoforms

The CA inhibitory ability of *R. coriaria* extract (RCE) was measured at different concentrations; 10, 25, 50, and 100 μg/ml as shown in (Table 2). Stopped-flow assay method was performed to estimate the inhibition and selectivity of sumac extracts against cytosolic CA isoforms (hCA I and II) and the membrane-associated CA isoforms (hCA IX and XII), respectively (Table 2). In addition, AZA and CQ as the reference inhibitors of CA isoforms were used in this experiment (Table 2). Compared with the effect of AZA and CQ drugs, sumac extract at doses of 10, 25, 50, and 100 μg/ml significantly produced a strong inhibition activity against all CA isoforms (Table 2). However, sumac extract at concentrations of 50 and 100 μg/ml showed higher inhibition activity compared with lower values (10 and 25 μg/ml) and the reference inhibitor controls (AZA and CQ) (Table 2).

**Table 2:** RCE inhibition activity to CA isoforms; hCA I, II, IX, and XII

RCE	ki (nM)^1^				SI^2^	
	hCA I	hCA II	hCA IX	hCA XII	hCA I/XII	hCA II/XII
10 μg/ml	31.8	8.5	11.5	4.9	42.8	3.1
25 μg/ml	29.5	6.7	9.3	2.8	46.8	8.4
50 μg/ml	21.8.	4.5	5.8	1.9	158.7	123.3
100 μg/ml	16.8	3.7	3.1	0.98	286.7	365.8
Standard CAIs						
AZM (μg/ml)	96.1	13.7	27.8	5.8	48.98	3.8
CQ (μg/ml)	98.6	15.6	36.7	11.3	259.7	136.71

AZM and CQ (Clioquinol), well-known CAIs, was used as a standard for comparison.

1 ki presented is the mean from three different assays; errors are in the range of ±5–10% of the reported values.

2 SI (selectivity index) is a ratio between the ki values observed for two hCA isoforms; low value index is indicative of weak selectivity.

The cytosolic isoform hCA I was inhibited by the sumac extract with ki values in the range of 16.8–31.8 nM. The most active sumac doses against hCA I were 50 and 100 μg/ml which showed the least ki values (21.8 and 16.8) compared with the moderate active doses; 10, 25 μg/ml with the higher ki values (31.8 and 29.5) respectively, However, all the sumac concentrations were more active than the reference CAI drugs (AZA and CQ) (Table 2).

Regarding the inhibitory activity of sumac against hCA II, all tested sumac concentrations were active with ki values in a range of 3.7–8.5 nM. Sumac at concentrations of 50 and 100 μg/ml were the most active with ki values (4.5 and 3.7) compared with lower sumac concentrations 10, 25 μg/ml which showed a moderate inhibition activity with ki values (8.5 and 6.7), respectively. Again all tested sumac concentrations being more active than AZA and CQ. Similarly, all sumac concentrations showed moderate to strong inhibition activity against both the tumor-associated target isoforms hCA IX (ki values; 3.1–11.5) and hCA XII (ki values; 0.98–4.9) (Table 2). The most active doses were at 50 and 100 μg/ml compared with the inhibition activity proposed to the reference drugs (AZA and CQ) (Table 2).

In this experiment, the selectivity index (SI) was calculated for each sumac concentration as hCA I/hCA XII and hCA II/hCA XII, respectively. All tested sumac extract concentrations showed high selectivity for the transmembranal tumor-associated isoform hCA XII than hCA I especially at higher dose 100 μg/ml with SI value more than 286. In addition, higher sumac concentrations; 50 and 100 μg/ml showed high selectivity for hCA XII than hCA II with SI values more than 123 and 365, respectively (Table 2). The selectivity of tested sumac concentrations was evaluated to reduce the unwanted side effects as the inhibition of the cytosolic isoforms hCA I and hCA II will lead to potential diuresis. The selectivity SI values of tested sumac at 100 μg/ml concentrations showed to be of more selectivity compared with both AZA and CQ CAIs (Table 2).

### Cytotoxic activity

**Figure 3:**
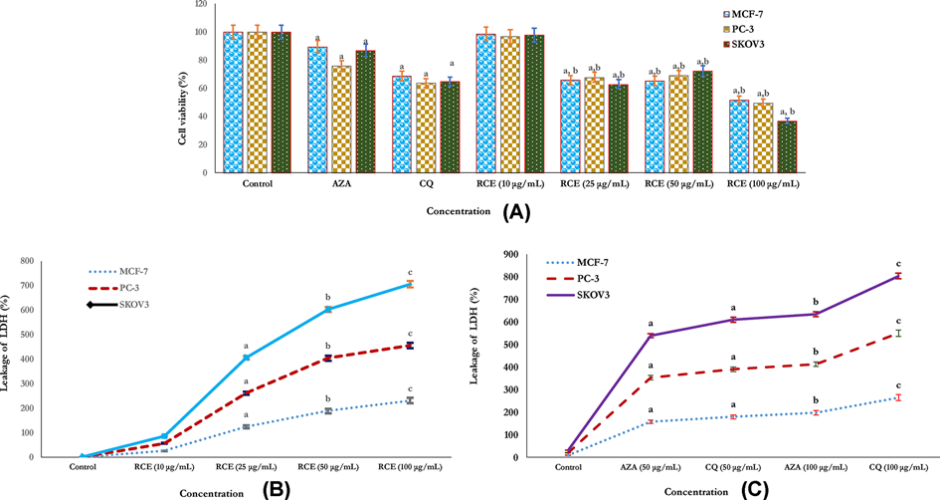
Determination of RCE cytotoxicity against human cancer cell lines Evaluation of cytotoxicity of RCE at doses of 10, 25, 50, and 100 μg/ml and related CAI enzyme isoforms inhibitors (Clioquinol and Acetazolamide) on human cancer cells MCF-7, PC-3, and SKOV3 for 24 h, as evaluated by MTT (**A**) and LDH assays (**B**,**C**). In MTT assay (A): the sumac extract (RCE) at higher doses of 25, 50, and 100 μg/ml significantly reduced the viability of the cancer cells; MCF-7, PC-3, and SKOV3 more than related inhibitors of CAI enzyme isoforms (Clioquinol and Acetazolamide). In LDH assays (B,C), LDH as a marker of cancer cell viability was significantly more released from the cancer cells in a dose-dependent manner form following treatment with sumac extract (RCE) (B) and CAI enzyme isoforms inhibitors (Clioquinol and Acetazolamide) (C), respectively. Each value represents the mean ±SE of three experiments. *n*=3; ^a^*P*<0.05, ^b^*P*<0.01, ^c^*P*<0.001 vs control (cancerous non-treated cell lines).

The cytotoxic activity of RCE at different concentrations on human cancer cells (MCF-7, PC-3, and SKOV3) was assessed by MTT and LDH assays as shown in (Figure 3A–C). RCE at higher doses 25, 50, and 100 μg/ml showed more adverse effects on human cancer cells SKOV3, PC-3, and MCF-7 in a dose-dependent manner compared with the reference CA drug inhibitors (AZA and CQ) and respective non-treated cancer cells (control) as shown in (Figure 3A). In addition, a variation in LDH activity was reported in the studied human cancer cells SKOV3, PC-3, and MCF-7 (Figure 3B,C). In cells treated with higher doses of sumac extract, the release of LDH enzyme was significantly higher in SKOV3 and PC-3 with lower levels of LDH in MCF-7 cell lines was reported which supports that the release of LDH in a dose-dependent manner. Also, the release of LDH was significantly increased at higher doses of (50 and 100 μg/ml) of CA drug inhibitors (AZA and CQ) which supports the mechanistic role of sumac as a potential CAI (Figure 3C). Thus it was confirmed that RCE was cytotoxic for the studied human cell lines and the result of the LDH test was in agreement with the finding of the MTT test (Figure 3B,C).

### Apoptosis in human cancer cells

**Figure 4:**
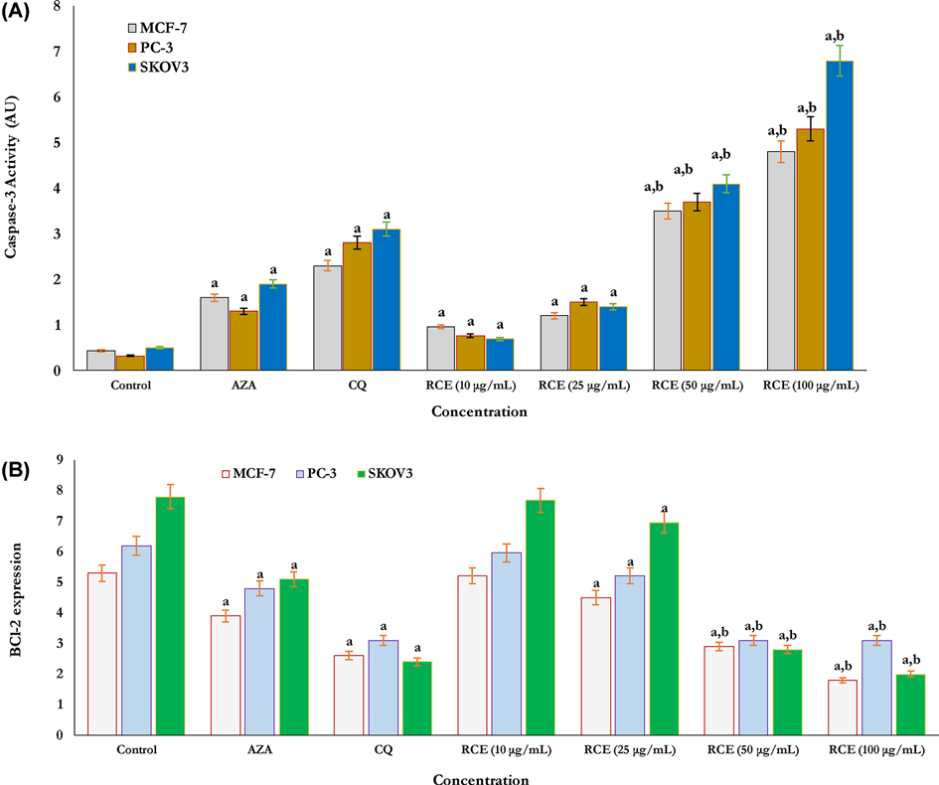
Evaluation of cancer cell apoptosis Cellular apoptosis was identified by estimating both caspase-3 activity (**A**) and Bcl-2 expression (**B**), respectively. Caspase-3 activity (A) significantly increased in cancer cells MCF-7, PC-3, and SKOV3 treated with RCE at doses of 50 and 100 μg/ml compared with related CAI enzyme isoforms inhibitors (Clioquinol and Acetazolamide) for 24 h. In addition, the expression of Bcl-2 protein (B), significantly reduced in cells treated with RCE at doses of 50 and 100 μg/ml compared with related CAI enzyme isoforms inhibitors (Clioquinol and Acetazolamide) for 24 h. The results signify that the anticancer activity of RCEs proceeds via a cellular apoptotic mechanism. Each value represents the mean ± SE of three experiments. ^a^*P*<0.05 (compared with control) and ^b^*P*<0.001 vs CAI standard control inhibitors (Clioquinol and Acetazolamide).

The activity of caspase-3 and Bcl-2 protein expression as apoptotic parameters were estimated in all sumac treated and non-treated human cancer cells as shown in (Figure 4A,B). The activity of caspase-3 was found more in SKOV3 cells than both PC-3 and MCF-7 cells, respectively (Figure 4A). The activity of caspase-3 was significantly more in 50 and 100 μg/ml RCE exposed SKOV3, PC-3, and MCF-7 cells (Figure 4A) as compared with CA drug inhibitors (AZA and CQ) exposed cells and non-treated control cells (Figure 4A), respectively. Also, the expression of Bcl-2 protein as an anti-apoptotic parameter was evaluated in RCE-treated and non-treated cancer cells (control) (Figure 4B).

In all studied human cancer cells, RCE-induced dose-dependent decline in Bcl-2 protein expression (Figure 4B) the results significantly compared with both CA drug inhibitors (AZA and CQ) exposed cells and non-treated cancer cells (control) as shown in Figure 4B. As in observation, there was more reduction in the expression of the anti-apoptotic protein Bcl-2 in SKOV3 cells treated with RCE at higher doses of 50 and 100 μg/ml than PC-3 and MCF-7 cells, respectively (Figure 4B).

## Discussion

The use of herbal-based therapy as an alternative strategy for chemotherapeutic drugs is highly appreciated in many human diseases [15–18]. Sumac (*R. coriaria L., Anacardiaceae)* one of the most commonly used spices is recommended in the treatment of several human diseases including cancer [12–14,19].

In the present study, potential nine bioactive compounds were estimated in the hydro-alcoholic extract of sumac. The characterized compounds were gallic acid, isohyperoside, dihydroxy methyl xanthone, β-Sitosterol-hexoside, α-Tocopherol, and linoleic acid. In addition, quercitrin, myricetin glucuronide, and myricetin rutinoside were identified as flavonoid derivatives in the extract of the sumac fruit. These compounds proposed to be potentially involved in the sumac effect on cancer cells and inhibition activity to CA isoforms of the studied human cancer cell lines; MCF-7, PC-3, and SKOV3.

The phytochemicals identified were significantly described recently in leaves and fruits of *R. coriaria* [49–51]. Quercitrin, myricetin rutinoside, dihydroxy methyl xanthone, β-Sitosterol-hexoside, α-Tocopherol, and linoleic acid are being reported in the fruits of *R. coriaria* [49–51]. In addition, sumac possesses and has shown to have a wide range of pharmacological properties, particularly antioxidant and cell-free radical scavenging activity, antiviral, and anticancer activities [52–54]. This is owing to its rich bioactive substances regarded in terms of phenolic compounds and flavonoids [50–54]. Moreover, active compounds present in sumac such as flavonoids, tannins and xanthons [20,21], reveal its potency as antiviral, antimicrobial, anticancer, antioxidant, and radical scavenging activities [15–19].

In the present study, sumac extract (RCE) at non-cytotoxic doses of 50 and 100 μg/ml strongly effect on the growth of the studied human cancer cell lines (MCF-7, PC-3, and SKOV3). In addition, the applied RCE doses showed a higher inhibitory effect with selectivity to overexpressed CA isoforms from cancer cells in comparison with the effect of the selected CA drug inhibitors (AZA and CQ). Moreover, both cytosolic isoforms (hCA I and II) and the membrane-associated isoforms (hCA IX and XII) were significantly inhibited at sumac concentrations; 50 and 100 μg/ml, respectively. Previously, it was reported that RCE significantly reduced cancer cell migration in cancer cell lines particularly BC cell lines [12,21,49].

In the present study, the selectivity of the sumac fruits extract (RCE) toward the expressed CA isoforms was estimated. The results showed that sumac extract at higher doses; 50 and 100 μg/ml significantly inhibited the overexpressed CA isoforms (hCA I; IIhCA; IX; and XII). Sumac showed high selectivity to hCA XII than hCA II with SI values more than 123 and 365, respectively. The selectivity SI values of tested sumac at 100 μg/ml concentrations showed to be more selective to expressed CA isoforms compared with traditionally used AZA and CQ CAIs. This potential selectively performed by sumac extract significantly helps in the reduction of unwanted side effects during therapeutic applications as the inhibition of the cytosolic isoforms hCA I and hCA II will lead to potential diuresis [23,24].

Several studies reported that phenols, polyphenols, and flavonoids present in plants were reported to be a competitive inhibitor of human (h) CA isoforms, particularly II (hCA II) isoform [55–57]. Also, several flavonoids showed promising inhibitory effects against human CA I, II, IV, VI and bovine CA III isoforms [58–61]. These studies support the presence of the catechol moiety in the polyphenols and flavonoids, which enhances the SI and inhibition activity to CA isoforms.

In the present study, the potential cytotoxic activity of sumac extract (RCE) was investigated in MCF-7, PC-3, and SKOV3 cells by using MTT and LDH assays. Cell viability was significantly reduced with the release of cellular LDH in higher quantities following exposure to different doses of RCE. More adverse effects on human cancer cells SKOV3, PC-3, and MCF-7 were reported at higher doses of RCE (25, 50, and 100 μg/ml) compared with the reference CA drugs inhibitors (AZA and CQ) and respective non-treated cancer cells (control). RCE at higher doses induced more cytotoxic effects on the SKOV3 than on the PC-3 and MCF-7 cells, respectively. This may be related to the anti-proliferative effects of the sumac exerted on treated cancer cells [13,62,63]. Previously, it was reported that rich secondary metabolites’ components present *R. coriaria* L. showed to responsible for the anticancer and growth inhibitory effects of sumac [21,64]. The co-similarity in the biological activity between sumac and the reference CA drugs inhibitors (AZA and CQ) as cytotoxic and as competitive inhibitors of human (h)CA isoforms might relate the phenolic properties of both the phytoconstituents of sumac and respective CA drugs inhibitors (AZA and CQ) which provide best phenolic inhibition activities against all CA isozymes [25–36].

Moreover, cellular apoptosis as an inhibition mode for the proliferation of cancer cells was investigated in the present study. The activation of caspase-3 and Bcl-2 anti-apoptotic protein as parameters of apoptosis were identified following exposure to RCE. RCE extract at higher doses of 50 and 100 μg/ml induced cellular apoptosis via increasing the activity of caspase-3 and the down-regulation of Bcl-2 respectively. Also, cellular apoptosis induced in all cells treated with CA drugs inhibitors (AZA and CQ). It was significant to note that SKOV3 cells were more susceptible to RCE extract than the PC-3 and MCF-7 cells. Our results matched with others who recently different extracts of *Rhus spp.* significantly inhibited the growth, proliferation, and viability of cancer cells by activating the apoptotic process via caspase-3 overexpression and the regulation of Bcl-2 anti-apoptotic protein [65–68]. However, this is the first evaluation of *in vitro* anticancer activity of from *R. coriaria* L. based upon inhibition activity and selectivity to CA isoforms which significantly overexpressed during the growth of some human cancer cell lines.

## Conclusion

The potential anticancer activity of *R. coriaria* L. has been fully discussed in the bases of inhibition activity and selectivity to CA isoforms expressed from different human cancer cell lines. The data showed that sumac at doses of 50 and 100 µM significantly inhibited the growth, proliferation, and viability of cancer cells by activating the apoptotic process via caspase-3 overexpression and the regulation of Bcl-2 anti-apoptotic protein. In addition, the strong inhibition activity and more selectivity of sumac towards CA isoforms hCA I and hCA II provide a potential use of this herbal plant as CAI in the treatment of cancer cells.

## Data Availability

All data generated or analyzed during the present study are presented in the manuscript. Please contact the corresponding author for access to data presented in the present study.

## Abbreviations

AZM/AZA: acetazolamide
BC: breast cancer
CA: carbonic anhydrase
CAI: CA inhibitor
CQ: Clioquinol (5-chloro-7-iodo-8-hydroxyquinoline)
LDH: lactate dehydrogenase
MTT: 3-(4,5-Dimethylthiazol-2-yl)-2,5-diphenyltetrazolium bromide
RCE: *Rhus coriaria* extract
RT: retention time
SI: selectivity index
